# 免疫检查点抑制剂相关内分泌不良反应的临床诊治建议

**DOI:** 10.3779/j.issn.1009-3419.2019.10.08

**Published:** 2019-10-20

**Authors:** 炼 段, 林杰 王, 晓燕 斯, 丽 张, 小伟 刘, 玥 李, 汉萍 王, 潇潇 郭, 佳鑫 周, 惠娟 朱, 力 张

**Affiliations:** 1 100730 北京，北京协和医学院内分泌科，卫键委内分泌重点实验室，中国医学科学院，北京协和医学院 Department of Endocrinology, Peking Union Medical College Hospital, Chinese Academy of Medical Science and Peking Union Medical College, 100730 Beijing, China; 2 100730 北京，北京协和医学院呼吸内科，卫键委内分泌重点实验室，中国医学科学院，北京协和医学院 Department of Respiratory, Peking Union Medical College Hospital, Chinese Academy of Medical Science and Peking Union Medical College, 100730 Beijing, China; 3 100730 北京，北京协和医学院检验科，卫键委内分泌重点实验室，中国医学科学院，北京协和医学院 Department of Laboratory, Peking Union Medical College Hospital, Chinese Academy of Medical Science and Peking Union Medical College, 100730 Beijing, China; 4 100730 北京，北京协和医学院眼科，卫键委内分泌重点实验室，中国医学科学院，北京协和医学院 Department of Ophthalmology, Peking Union Medical College Hospital, Chinese Academy of Medical Science and Peking Union Medical College, 100730 Beijing, China; 5 100730 北京，北京协和医学院消化内科，卫键委内分泌重点实验室，中国医学科学院，北京协和医学院 Department of Gastroenterology, Peking Union Medical College Hospital, Chinese Academy of Medical Science and Peking Union Medical College, 100730 Beijing, China; 6 100730 北京，北京协和医学院心内科，卫键委内分泌重点实验室，中国医学科学院，北京协和医学院 Department of Cardiology, Peking Union Medical College Hospital, Chinese Academy of Medical Science and Peking Union Medical College, 100730 Beijing, China; 7 100730 北京，北京协和医学院风湿免疫科，卫键委内分泌重点实验室，中国医学科学院，北京协和医学院 Department of Rheumatology, Peking Union Medical College Hospital, Chinese Academy of Medical Science and Peking Union Medical College, 100730 Beijing, China

**Keywords:** 免疫检查点抑制剂, 免疫相关不良反应, 内分泌系统, Immune checkpoint inhibitors, Immune-related adverse events, Endocrine system

## Abstract

免疫检查点抑制剂（immune checkpoint inhibitors, ICIs）作为一类新型抗肿瘤药物，已经在多种恶性肿瘤治疗中表现出显著的疗效。由于ICIs特定的作用目标和机制，可引起自身免疫和炎症效应，称为免疫相关不良反应（immune-related adverse effects, irAEs）。与传统疗法的不良反应机制不同，irAEs表现隐匿且不固定，有些严重不良反应将迫使患者中止治疗，甚至影响患者生存。因此，随着ICIs在临床上的广泛应用，临床医生需要充分认识此类药物治疗可能产生的不良反应以及合理的治疗策略，以提高接受ICIs治疗患者的生存率和治疗效果。本文将对ICIs引起的内分泌系统不良反应的发生率、临床表现、诊断和处理分别进行阐述。

## Abstract (en)

As a new type of anti-cancer drugs, immune checkpoint inhibitors (ICIs) have shown remarkable therapeutic effects in many malignant tumors. By virtue of their targets and mechanisms of action, ICIs can cause autoimmune and inflammatory effects, termed immune-related adverse events (irAEs). Unlike traditional therapies, irAEs are latent and unstable. Severe adverse reactions will force patients to discontinue treatment and even affect their survival. Therefore, with the wide application of ICIs in clinical practice, clinicians need to fully understand the possible adverse reactions of such drugs and appropriate treatment strategies, in order to improve the survival rate and treatment effect of patients receiving ICIs. In this article, we review the incidence, clinical features, diagnosis and treatment of immune-related endocrine events that may occur with administration of ICIs.

内分泌系统不良反应多为迟发型，中位发生时间为用药开始后9周（5周-36周），最常见不良反应为垂体炎和甲状腺功能异常，也可累及肾上腺、胰腺和甲状旁腺，表现为原发性肾上腺皮质功能减退症、自身免疫性糖尿病和甲状旁腺功能减退症。ICI诱发垂体炎的发生时间是6周-14周，甲状腺功能异常的发生时间通常是4周-7周^[1]^。内分泌功能的恢复往往需要较长的时间，如果不及时治疗甚至危及生命，因此需要早期识别并给予适当处理。

## 1 垂体炎

### 1.1 发生率

接受ipilimumab伊匹木单抗治疗的患者垂体炎的发生率为1.5%-17%，而nivolumab纳武利尤单抗诱发垂体炎的比率仅有0.6%-1.5%，联合药物治疗导致垂体炎的发生率较高，伊匹木单抗联合纳武利尤单抗或pembrolizumab帕博利珠单抗治疗的发生率分别为4.0%-12.8%和9.1%^[2]^。既往研究发现，垂体细胞上异位表达细胞毒性T淋巴细胞相关抗原4（cytotoxic T lymphocyte-associated antigen 4, CTLA-4），CTLA-4单抗与抗原结合后可以激活经典补体级联反应，导致Ⅱ型超敏反应引起垂体炎的发生^[3]^。

### 1.2 临床表现

ICI诱发的垂体炎可以引起全垂体功能减退或孤立垂体前叶激素缺乏，并且伴或不伴垂体增大，仅有少数患者会出现垂体增大导致的占位相关症状。最常见的临床表现有头痛、乏力，其他表现包括记忆力下降、视力损害、眩晕、厌食、恶心、腹泻、心动过速、低血压、性功能下降、闭经等。尽管大多数患者可以出现多种垂体激素缺乏，但是继发性甲状腺功能减退、低促性腺激素性性腺功能减退和继发性肾上腺皮质功能减退更为常见，发生率分别为93%、86%和75%，而生长激素（growth hormone, GH）和催乳素（prolactin, PRL）较少受累，与其他自身免疫性垂体炎相比，尿崩症并不常见^[4, 5]^。值得注意的是，有些垂体炎的患者可以发生肾上腺危象，甚至危及生命，典型表现有低血压或休克、发热、厌食、恶心、呕吐、意识障碍、昏迷和电解质紊乱（如低钠血症、高钾血症），这种情况需要与严重的脓毒血症相鉴别。

### 1.3 诊断

由于垂体炎患者的临床表现并不特异，而且与肿瘤进展引起的症状相类似，因此需要与感染、脑转移等其他病因相鉴别，垂体功能的评价至关重要。当患者出现下列症状时：①新发中度疲劳或头痛；②新发恶心、呕吐或腹泻；③眩晕、体位性低血压或低钠血症；④血流动力学不稳定，需要考虑有垂体炎可能，应及时筛查生化指标（包括血电解质、空腹血糖），同时评价垂体功能，包括：①垂体-肾上腺轴，清晨空腹促肾上腺皮质激素（adrenocorticotropic hormone, ACTH）和血皮质醇；②垂体-甲状腺轴，促甲状腺激素（thyrotropin, TSH）和游离甲状腺素（free thyroxine, FT_4_）；③垂体-性腺轴，卵泡刺激素（follicle stimulating hormone, FSH）、黄体生成素（luteinizing hormone, LH）和雌二醇（estradiol, E_2_）或睾酮（testosterone, T）；④PRL、GH和胰岛素样生长因子1（insulin-like growth factor-1, IGF-1）。如果患者出现口渴、多尿、多饮，还需要查同步的血钠、血渗透压、尿渗透压和尿比重。临床症状发生之前即可以出现垂体增大和垂体柄增粗，因此临床上怀疑垂体炎的患者需要做鞍区核磁了解垂体增大的程度，明确有无视神经受压，同时除外脑转移病变。由于伊匹木单抗诱发垂体炎的比率较高，当患者接受伊匹木单抗治疗时，需要重视垂体功能的评估。

### 1.4 治疗

若患者临床上出现血容量不足、低血压、低钠血症、高钾血症或低血糖时，需要考虑肾上腺皮质功能减退症，若血皮质醇降低，ACTH无明显升高，可诊断继发性肾上腺皮质功能减退。回顾性研究发现，超生理剂量糖皮质激素治疗与替代剂量治疗相比，并不能更好的改善临床症状及缩短垂体功能恢复的时间，同时使用较大剂量激素有增加感染、高血糖的发生风险，因此治疗推荐采用氢化可的松10 mg/d-30 mg/d，分次给药^[6]^。如果发生肾上腺危象、严重低钠血症或严重头痛，需停止免疫调节治疗，并且短期给予大剂量糖皮质激素，如静脉给予氢化可的松100 mg，每8小时1次^[4, 5]^。

若甲功结果提示FT_4_降低，同时TSH降低或在不适当的正常参考范围内，可诊断继发性甲状腺功能减退症，需要给予左甲状腺素钠（L-T4）替代治疗，推荐起始剂量0.8 μg/kg/d，年龄 > 60岁或合并心血管疾病的患者需要从小剂量起始（12.5 μg/d-25 μg/d），4周后复查甲功，依据FT_4_水平滴定药物剂量。在L-T4替代治疗前，首先要除外继发性肾上腺皮质功能减退，如果两者同时存在，必须先补充糖皮质激素，这是因为甲状腺激素会促进糖皮质激素的清除，仅补充L-T4会加重肾上腺皮质功能不全，甚至诱发肾上腺危象^[4, 5]^。

如果没有治疗禁忌，男性患者可以补充睾酮。由于雌激素与某些恶性肿瘤和静脉血栓的发生风险相关，绝经前女性患者需慎用雌激素替代治疗。恶性肿瘤患者应禁用生长激素治疗^[4]^。

给予合理替代治疗的患者通常不需要停止免疫调节治疗，仅在患者出现垂体增大导致占位症状（如视力受损和头痛）和肾上腺危象时需要暂时停用免疫治疗^[2]^。回顾性研究发现，63%-85%患者甲状腺功能可以恢复正常，约一半男性患者性腺功能恢复，但是肾上腺皮质功能却少有恢复。ICI诱发的垂体炎患者中，垂体增大几乎均可以恢复，平均恢复时间是15周（2周-27周）^[5, 6]^。

## 2 甲状腺功能异常

### 2.1 发生率

与垂体炎不同，ICI诱发的甲状腺功能异常更常见于抗PD-1单抗，平均发生时间是在首次治疗后6周。一项荟萃研究结果显示，抗CTLA-4、抗PD-1和抗PD-L1诱发甲状腺功能减退的比率分别为3.8%、7.0%和3.9%，纳武利尤单抗联合伊匹木单抗引起甲状腺功能减退的比率高达13.2%。抗CTLA-4、抗PD-1和抗PD-L1诱发甲状腺功能亢进的比率分别为1.7%、3.2%和0.6%，纳武利尤单抗联合伊匹木单抗引起甲亢的比率为8.0%^[7]^。

ICI诱发甲状腺功能异常的发生机制尚不明确。既往研究发现*CTLA*-*4*基因多态性与自身免疫性甲状腺疾病（如Graves病和桥本甲状腺炎）有关。另有临床研究^[8]^显示，ICI诱发的甲状腺功能异常可能与细胞毒性T淋巴细胞介导的甲状腺组织破坏有关。尽管有些学者认为，大多数ICI诱发甲状腺功能异常的患者存在甲状腺自身抗体，但是两者之间的相关性尚无明确结论，需要前瞻性研究进一步证实。

### 2.2 临床表现

由于常规检测甲功，ICI诱发的甲功异常通常在早期即被诊断，患者一般没有症状或症状轻微，大多数因症状来就诊的患者首发表现是甲状腺毒症，如心动过速、多汗、腹泻和体重下降。甲状腺功能减退的临床表现包括疲劳、乏力、便秘、畏寒、皮肤干燥和体重增加。尽管大多数患者临床表现轻微，极少数患者需要停止ICI治疗或起始免疫抑制治疗，但是已有ICI治疗诱发甲状腺危象或严重甲状腺功能减退的病例报道。

### 2.3 诊断

甲状腺功能检测有助于判断患者的甲功状态：①如果FT_4_降低，伴TSH升高，可诊断原发性甲状腺功能减退；②如果FT_4_升高，TSH降低时，诊断甲状腺毒症；③如果FT_4_下降，TSH降低或在正常范围内，需考虑中枢性甲状腺功能减退，应进一步评价垂体功能，明确是否为ICI诱发的垂体炎。由于ICI诱发的甲功异常发生在治疗后的较短时间内，有学者建议在ICI治疗前和开始治疗的前5个周期的每次用药前均应该检测甲功，此后每3个月复查甲功，每次评估病情需要关注甲功异常的相关症状。此外需要注意的是，增强CT检查使用的含碘造影剂可能会影响甲功检测结果，并且使患者无法再进行吸碘率检查鉴别甲状腺炎（吸碘率降低）或Graves病（吸碘率增加）引起的甲状腺毒症。如果患者出现甲状腺相关眼病或甲状腺肿，应该筛查TSH受体抗体，对于近期未曾使用含碘造影剂的患者建议做吸碘率检查协助诊断。

### 2.4 治疗

发生甲功异常的患者通常不需要停止ICI治疗，如果是原发性甲状腺功能减退，需要补充L-T4，具体治疗方案参见继发性甲状腺功能减退。如果患者表现为甲状腺毒症（如心率 > 100 bpm且无低血压），可以短期使用β受体阻滞剂改善症状。由于大多数甲状腺毒症患者会逐渐进展为甲状腺功能减退状态（平均发生时间为6周），建议每2周-3周复查甲功，如果患者出现甲状腺功能减退，应停用β受体阻滞剂，并补充L-T4维持甲功正常。当伴有心血管疾病风险的老年患者发生严重的甲状腺毒症时，可短期使用大剂量糖皮质激素（口服泼尼松1 mg/kg/d，1周-2周）。如果TSH受体抗体阳性或甲状腺吸碘功能亢进，需给予抗甲状腺药物治疗（如甲巯咪唑）^[4]^。

## 3 原发性肾上腺皮质功能减退

### 3.1 发生率

文献报道自身免疫性肾上腺炎的发生率仅有0.7%（43/5831），在联合药物治疗患者中的发生率4.2%，有学者推测共同阻滞CTLA-4和PD-1/PD-L1在肾上腺炎的发生中起着重要的作用^[7]^。

### 3.2 临床表现和治疗

当患者出现以下症状或体征时需要考虑肾上腺皮质功能减退：乏力、血容量不足、低血压、低钠血症、高钾血症、低血糖、发热、腹痛、皮肤色素沉着、体重下降等。需要关注患者生命体征，同步检测清晨空腹血皮质醇和ACTH，当血皮质醇降低，ACTH升高，诊断原发性肾上腺皮质功能减退症；若血皮质醇和ACTH均降低，符合继发性肾上腺皮质功能减退症。若患者无明显临床表现，可继续免疫调节治疗，给予氢化可的松10 mg/d-30 mg/d，分2次口服；若患者出现相关症状（如乏力、厌食）但未出现血流动力学不稳定时，需停用免疫调节治疗，并给予氢化可的松10 mg/d-30 mg/d，分2次口服；若患者发生肾上腺危象（如低血压休克、脱水、意识障碍、腹痛、呕吐、发热等），应停止免疫调节治疗，静脉给予氢化可的松100 mg每8小时1次；若诊断原发性肾上腺皮质功能减退，还需补充盐皮质激素（氟氢可的松0.05 mg/d-0.2 mg/d）。由于肾上腺皮质功能减退很难恢复，患者通常需要长期激素替代治疗，应该对患者和家属进行宣教，特别是在应激状态时需及时增加糖皮质激素剂量（通常是替代剂量的2倍-3倍）^[4]^。

## 4 自身免疫性糖尿病

国外学者回顾性分析了24例ICI诱发的自身免疫性糖尿病，50%（11/22）患者存在自身抗体阳性，出现临床表现的平均时间是在起始治疗后的8.5周（1周-12个月），75%（18/24）患者以糖尿病酮症酸中毒（diabetic ketoacidosis, DKA）起病，所有患者糖化血红蛋白均有升高，10例患者C肽水平明显降低或检测不出，提示胰岛β细胞功能衰竭速度较快^[9]^。一旦诊断，需要立刻起始胰岛素治疗，给予基础联合餐时胰岛素，必要时可给予皮下胰岛素泵控制血糖。如果患者出现DKA，应及时给予补液、持续胰岛素输注，同时关注血气pH值和电解质紊乱，需要停止免疫调节治疗直到血糖获得有效控制^[4]^。

## 5 甲状旁腺功能减退

国外学者^[10]^在2017年首次报道了ICIs诱发的甲状旁腺功能减退，1例73岁转移性黑色素瘤男性患者，接受伊匹木单抗联合纳武利尤单抗治疗后出现严重低钙血症（血钙5 mg/dL）和高磷血症（血磷6.6 mg/dL），同步甲状旁腺素明显降低（< 1 pg/mL），随后患者发生甲状腺毒症，并逐渐出现原发性甲状腺功能减退。随访至4个月时，患者的甲状腺和甲状旁腺功能均未能恢复，需要接受L-T4、钙剂和骨化三醇替代治疗。

## 6 总结

ICI诱发的内分泌功能异常的发病机制和不良反应发生的预测因素还有待更多的临床研究进一步证实，临床医生和患者需要了解的是，多数内分泌功能异常无法恢复，需要多学科协作制定个体化治疗方案，并且患者应该接受长期随访。有些内分泌急症（如肾上腺危象）如果不能早期识别并给予适当处理，可能危及患者生命，因此应该重视内分泌功能的评估，并且需要内分泌专科医生参与患者的诊治和随访。
